# Brote de toxoplasmosis pulmonar en militares inmunocompetentes

**DOI:** 10.7705/biomedica.7728

**Published:** 2026-03-02

**Authors:** Raúl Reyes-Toledo, Laura Culma-Roa, María José López, Jairo Enrique Pérez, David Suárez-Silva

**Affiliations:** 1 Servicio de Medicina Interna, Hospital Militar Central, Bogotá, D. C., Colombia Hospital Militar Central Bogotá D. C Colombia; 2 Programa de Medicina Interna, Universidad Militar Nueva Granada, Bogotá, D. C., Colombia Universidad Militar Nueva Granada Universidad Militar Nueva Granada Bogotá D. C Colombia; 3 Servicio de Infectología, Hospital Militar Central, Bogotá, D. C., Colombia Hospital Militar Central Bogotá D. C Colombia

**Keywords:** toxoplasmosis, miocarditis, técnicas de genotipificación, técnicas de laboratorio clínico, personal militar, Toxoplasmosis, myocarditis, genotyping techniques, clinical laboratory techniques, military personnel

## Abstract

La toxoplasmosis es una infección zoonótica frecuente con diversos mecanismos de transmisión y distribución amplia a nivel mundial. Su probabilidad de generar enfermedad está relacionada con la alteración del sistema inmunológico y la virulencia del parásito. Se presentan cuatro pacientes sin inmunosupresión conocida, procedentes de un área selvática de Colombia y con diagnóstico de toxoplasmosis confirmado por serología y con compromiso pulmonar, uno de los cuales desarrolló falla respiratoria hipoxémica y miocarditis. Este brote de toxoplasmosis se presentó en personal militar después de su exposición a esta zoonosis en la Amazonia colombiana durante un ejercicio de entrenamiento.

Se describen el proceso diagnóstico, las manifestaciones clínicas y el tratamiento, y se discute la importancia de las variantes genotípicas del parásito.

## Abstract (en)

Toxoplasmosis is a common zoonotic infection with various transmission mechanisms and wide distribution worldwide. Its probability of causing disease is related to alterations in the immune system as well as the parasite's virulence.

This study presents four cases of patients from rainforest areas in Colombia who did not have any known immunosuppression but do have a diagnosis of toxoplasmosis with pulmonary involvement, confirmed by serology. One of them also developed hypoxemic ventilatory failure and myocarditis. This outbreak of toxoplasmosis occurred in military personnel after coming into contact with common sources of exposure in the Colombian Amazon during a military training exercise.

This article includes the diagnostic process, clinical manifestations, treatment, and importance of the parasite's genotypic variants.

La toxoplasmosis es una infección zoonótica de distribución mundial causada por el parásito intracelular obligado *Toxoplasma gondii.* Su prevalencia está determinada por múltiples factores, incluidos los hábitos alimentarios, el nivel socioeconómico, la edad, el estado inmunológico y las condiciones ambientales ^1^.

Dada su amplia distribución, el contacto con el parásito es común a lo largo de la vida. No obstante, la progresión a enfermedad depende de la integridad del sistema inmunológico y de la virulencia del parásito ^1^^,^^2^. En las personas inmunocompetentes, la infección suele ser asintomática o cursar con síntomas leves similares a los de un cuadro gripal, a veces acompañada de adenomegalia aislada. Por el contrario, en personas inmunosuprimidas, como pacientes con VIH y recuentos de linfocitos CD4 menor de 100 células/μl sin profilaxis, se observa reactivación en cerca del 30 % de los casos, con afectación frecuente del sistema nervioso central ^3^.

En Suramérica, la virulencia del parásito adquiere especial relevancia por la presencia de cepas genotípicas atípicas asociadas con mayor gravedad clínica y mortalidad ^4^. En Colombia, son escasos los reportes de toxoplasmosis grave en pacientes inmunocompetentes, definida como aquella con afectación, por lo menos, de tres órganos (pulmones, sistema nervioso central y corazón), curso prolongado (de tres o más meses) o desenlace fatal. La mayor parte de la información disponible proviene de las estrategias de salud pública orientadas a la prevención de la toxoplasmosis congénita y de los estudios sobre toxoplasmosis ocular. En estos últimos, se ha reportado una prevalencia de hasta el 10 % de cicatrices retinocoroideas sugestivas de infección previa, asociadas con seropositividad. Además, entre el 50 y el 60 % de las mujeres embarazadas presentan anticuerpos contra *T. gondii,* lo cual evidencia una amplia exposición en la población general ^5^^-^^9^.

En este contexto, se presenta una serie de cuatro casos de toxoplasmosis aguda con compromiso pulmonar en pacientes inmunocompetentes, procedentes de la selva amazónica colombiana.

## Presentación de casos

El caso índice correspondió a un miembro masculino del Ejército Nacional, de 28 años de edad y procedente de Leticia (Amazonas), que ingresó al Hospital Militar Central tras dos semanas de síntomas caracterizados por fiebre, mialgias, artralgias y cefalea, seguidos de diarrea acuosa, tos no productiva y disnea progresiva. Los síntomas se iniciaron durante un curso militar en una zona selvática del Amazonas. Previamente, había recibido gentamicina, dipirona y dexametasona intramuscular, con alivio parcial de los síntomas, motivo por el cual fue remitido al hospital.

Como antecedentes epidemiológicos relevantes, se documentó el consumo de carne de cerdo mal cocida días antes del inicio de los síntomas, junto con la ingestión de agua no tratada y malas condiciones sanitarias durante la actividad militar. El paciente no tenía antecedentes personales, farmacológicos, quirúrgicos, alérgicos ni de exposición conocidos, antes del inicio de los síntomas. El examen físico de ingreso reveló fiebre, taquipnea (28 respiraciones por minuto), taquicardia (113 latidos por minuto) y necesidad de oxígeno suplementario. En la auscultación pulmonar, se encontró disminución de la intensidad de los ruidos respiratorios en ambas bases pulmonares. No presentaba adenomegalias ni visceromegalias palpables.

**Figura 1:**
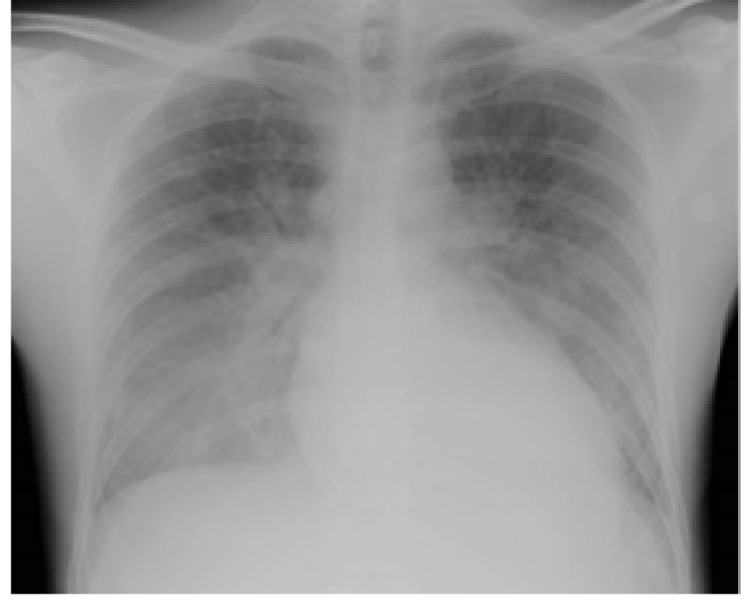
Caso 1. Radiografía frontal de tórax: opacidades con características reticulonodulares, predominantemente en las bases pulmonares y en el campo pulmonar derecho

Los exámenes de laboratorio de ingreso mostraron anemia leve (hemoglobina 11 g/dl) con volúmenes normales, aumento de las transaminasas (AST = 119 U/L y ALT = 131 U/L), sin alteraciones de la función renal, ni trastorno electrolítico; los gases arteriales mostraron alcalemia respiratoria sin hipoxemia y un trastorno leve de la oxigenación. En los estudios iniciales, se descartó malaria y la serología fue negativa para dengue. La radiografía de tórax (figura 1) evidenció opacidades reticulonodulares en ambas bases pulmonares y un pequeño derrame pleural izquierdo. Inicialmente, fue hospitalizado por parte del Servicio de Medicina Interna con diagnóstico de neumonía adquirida en la comunidad y se inició antibioticoterapia con ampicilina-sulbactam.

Al día siguiente, ingresaron dos militares más, de 25 y 28 años de edad, que se encontraban en el mismo curso militar, quienes presentaban iguales características clínicas y antecedentes de exposición que las del caso índice. Además, por medio de la información aportada por los pacientes, se logró identificar un cuarto caso atendido en otra institución, que se inició con un síndrome febril acompañado de alteración del estado de conciencia y miocarditis documentada, el cual progresó a falla respiratoria.

**Figura 2:**
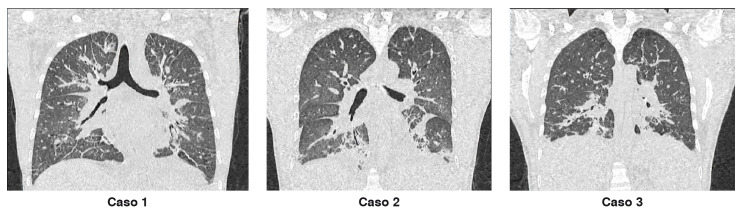
Tomografia computarizada de alta resolución de los tres primeros casos

Se analizaron las características clínicas y se estudiaron las principales causas de síndrome febril agudo de pacientes procedentes de áreas endémicas para enfermedades transmitidas por vectores. En conjunto con el Servicio de Infectología, y con base en la exposición común, el patrón clínico y el comportamiento en brote de la enfermedad, se planteó el diagnóstico presuntivo de toxoplasmosis con compromiso pulmonar en pacientes inmunocompetentes. Se inició tratamiento con trimetoprim-sulfametoxazol intravenoso a una dosis de 5 mg/kg cada 12 horas y se solicitaron los estudios serológicos para *T. gondii.* Las tomografías de tórax mostraron los hallazgos indicativos de la infección por toxoplasma descritos en la literatura: opacidades reticulonodulares, engrosamiento septal y áreas en vidrio esmerilado con escaso derrame pleural (cuadro 1 y figura 2).

**Cuadro 1:** Hallazgos en la tomografía de tórax

Caso	Pulmonar	Mediastinal	Pleural
1	Opacidades reticulonodulares, engrosamiento septal y áreas en vidrio esmerilado	Ninguno	Derrame pleural de predominio derecho
2	Opacidades reticulonodulares, engrosamiento septal y áreas en vidrio esmerilado	Ninguno	Derrame pleural escaso
3	Opacidades reticulonodulares, engrosamiento septal y áreas en vidrio esmerilado	Adenopatías mediastinales	Derrame pleural escaso
4	Opacidades con consolidación en ambos lóbulos superiores con engrosamiento septal	Ninguno	Derrame pericárdico leve y derrame pleural bilateral moderado

**Figura 3:**
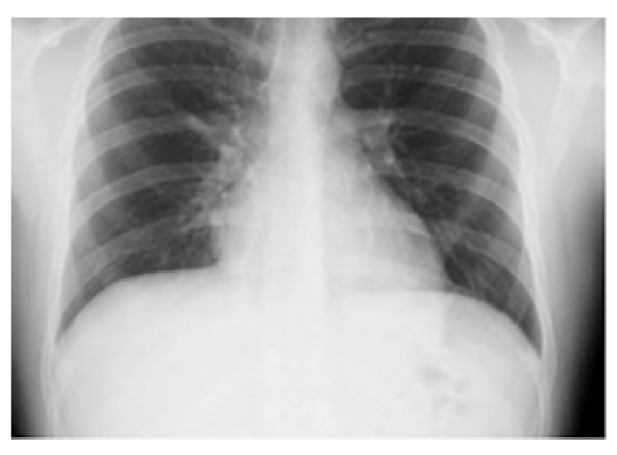
Caso 1. Radiografia frontal de tórax después de 21 días de tratamiento antibiótico con trimetoprim-sulfametoxazol

La serología fue positiva para IgM e IgG contra *T. gondii* en los cuatro casos, con pruebas de avidez baja, confirmándose la infección reciente y la sospecha diagnóstica (cuadro 2). Los pacientes completaron 21 días de tratamiento con trimetoprim-sulfametoxazol, con la resolución de los síntomas y de los cambios imagenológicos pulmonares (figura 3).

**Cuadro 2:** Resultados de la serologia

Estudio de laboratorio	Caso 1	Caso 2	Caso 3	Caso 4
IgM *ant\-Toxoplasma* (UI/ml)*	27,0	227	73,1	120
IgG anti-Toxoplasma (UI/ml)**	31,14	20,5	25,09	242
Test de avidez (%)***	6,8	19,25	9,7	-

* ELISA, valores de referencia: negativo < 0,8 UI/ml; indeterminado 0,8 a 1,0 UI/ml; positivo > 1,0 UI/ml

** ELISA, valores de referencia: negativo < 0,8 UI/ml; indeterminado 0,8 a 1,0 UI/ml; positivo > 1,0 UI/ml

*** ELISA, valores de referencia: baja < 20 %; intermedia 20 a 30 %; alta > 30 %

### Consideraciones éticas

Se obtuvo el consentimiento informado de los pacientes para la publicación de la presente serie de casos. Esta investigación se realizó conforme a los principios éticos establecidos en la Declaración de Helsinki de la Asociación Médica Mundial, el Informe Belmont, las directrices del Consejo de Organizaciones Internacionales de las Ciencias Médicas (CIOMS) y las normas colombianas vigentes (Resolución 8430 de 1993), que clasifican este tipo de estudio como una investigación sin riesgo. Por tal motivo, no se requirió la aprobación del Comité de Ética del Hospital Militar Central.

## Discusión

*Toxoplasma gondii* es un parásito intracelular perteneciente a la familia Apicomplexa, caracterizado por presentar organelos en el extremo (apical) que utiliza para penetrar en la célula huésped. Su ciclo de vida involucra huéspedes definitivos (felinos) e intermediarios (mamíferos, aves, humanos), con fases sexuales en los gatos y asexuales en los otros. Se inicia cuando el gato ingiere ooquistes o quistes tisulares, liberando taquizoitos que forman quistes de bradizoitos en los tejidos. Los humanos se infectan por ingerir carne cruda, agua contaminada o entrar en contacto con heces felinas, formándose quistes que persisten de por vida y se reactivan si disminuye la capacidad inmunológica ^10^^-^^12^; también, pueden transmitirse de la madre al feto o por medio de transfusiones ^13^.

El desarrollo de los sintomas y su gravedad están condicionados por factores relacionados con el huésped y el parásito. La infección puede ser congénita y las deficiencias inmunitarias adquiridas del huésped -como las presentes en la infección por HIV, los receptores de trasplantes o los cánceres hematológicos, o con el uso de medicamentos inmunosupresores-permiten la reactivación de una infección latente. Se puede comprometer cualquier órgano, especialmente los ojos y el sistema nervioso central, y se relaciona con importantes tasas de morbilidad y mortalidad ^14^. En condiciones de inmunocompetencia, la virulencia de la cepa involucrada en la infección y el efecto del inóculo son los principales factores relacionados con el desarrollo de enfermedad ^15^.

En el mayor estudio realizado sobre la diversidad genética de las poblaciones de parásitos de la especie *Toxoplasma,* se encontraron 11 haplotipos, cuatro de ellos exclusivos de Suramérica y que confirman la divergencia geográfica entre el norte y el sur. En Suramérica, la diversidad genética de *Toxoplasma* spp. es mucho mayor que en otros continentes. Además, las cepas suramericanas poseen con mayor frecuencia alelos virulentos como ROP18 o ROP16. En los estudios colombianos, se ha demostrado que estas cepas causan formas clinicas más graves pues inhiben el efecto protector del IFN-v. Esto explicaria la mayor gravedad de las manifestaciones clinicas de los casos colombianos, en comparación con los de otros continentes ^16^^,^^17^

En ciertas regiones de Suramérica, especialmente en la selva amazónica, algunas cepas atipicas reflejan fenómenos de recombinación genómica y han logrado difundirse a los animales peridomésticos, lo cual ha generado brotes en poblaciones en contacto con fuentes contaminadas, como el agua y las carnes mal cocidas ^4^^,^^18^^,^^19^.

Por ejemplo, Carme *et al.* (2002) describieron casos de toxoplasmosis multivisceral en personas inmunocompetentes, enmarcados inicialmente en un sindrome infeccioso inespecifico, con desarrollo posterior de afectación principalmente pulmonar (14 de 16 casos) y con reacción favorable al tratamiento antibiótico. En los análisis genéticos por microsatélites de las cepas involucradas, se evidenció un genotipo atipico multilocus, con un alelo único para esta región, por lo cual se planteó que la enfermedad grave en humanos puede estar relacionada con una pobre adaptación del huésped humano a estas cepas muy virulentas que circulan en el ciclo silvestre de dicha región ^20^.

En los análisis genéticos posteriores de los brotes de toxoplasmosis aguda en la misma área geográfica, se demostró una misma cepa atipica (5 de 11 casos), causante de manifestaciones clinicas diversas, desde infecciones leves hasta toxoplasmosis diseminada con gran riesgo de mortalidad. Esto puede explicarse por las variaciones en la susceptibilidad genética del huésped a estas cepas silvestres de toxoplasma y por diferencias en el inóculo ^21^.

La presentación clinica más habitual en la toxoplasmosis multivisceral es un cuadro clinico de fiebre inespecifica, que puede confundirse con otras causas del sindrome febril agudo del trópico y que, 10 a 15 dias después, puede acompañarse principalmente de afectación pulmonar. En un tercio de los casos, se trata de una forma grave que requiere iniciar rápidamente el tratamiento contra *T. gondii* pues, de lo contrario, puede ser letal ^20^.

En Colombia, en el 2009, se reportó el primer y mayor brote epidémico de toxoplasmosis aguda en pacientes inmunocompetentes. Se presentó en un grupo de 18 hombres pertenecientes a las fuerzas militares, todos con un nexo epidemiológico de condiciones sanitarias inadecuadas en la región selvática. Se confirmó mediante inmunofluorescencia indirecta de anticuerpos IgG anti- *Toxoplasma,* con titulos iguales o superiores a 1:1024. Principalmente, se presentó compromiso pulmonar y gastrointestinal; en uno de estos casos, hubo compromiso miopericárdico grave, lo cual se atribuyó a una probable infección por cepas silvestres de *T. gondii*^22^.

Cortés y Aguirre, en el 2018, reportaron un caso mortal de toxoplasmosis aguda por genotipos silvestres, en un paciente inmunocompetente con deterioro neurológico progresivo y falla multiorgánica. En la autopsia, se encontró compromiso miocárdico, cerebral y del músculo esquelético, por formas quisticas de *T. gondii* que fueron confirmadas mediante técnicas de inmunohistoquimica ^23^.

Existen diferentes métodos de confirmación diagnóstica para la infección por *T. gondii,* entre ellos, la identificación microscópica de formas parasitarias en muestras de tejido, el análisis de secuenciación genética por biologia molecular, como PCR de liquidos corporales o tejido, o la inoculación de la infección en modelos animales. Sin embargo, estas herramientas diagnósticas no son prácticas y carecen de disponibilidad suficiente para utilizarse de forma sencilla en la práctica clinica actual ^24^.

Por esta razón, el diagnóstico de la toxoplasmosis se basa habitualmente en la identificación serológica de la infección mediante la determinación de anticuerpos, la cual puede realizarse mediante diversas técnicas, cada una con rendimientos diagnósticos diferentes ^25^^,^^26^. Los anticuerpos IgG comienzan a aumentar entre una y tres semanas después de la infección, con un pico entre la sexta y la octava semanas, lo cual refleja un contacto previo con el parásito. La detección de anticuerpos IgM sugiere una infección reciente o aguda. Sin embargo, existe un gran porcentaje de casos en los cuales los títulos de anticuerpos IgM pueden persistir por largos periodos, algunas veces por varios años; además, esto se suma a la gran frecuencia de falsos positivos, lo cual limita su utilidad diagnóstica para confirmar la infección aguda ^27^^,^^28^.

Por tales motivos, se han desarrollado métodos complementarios, como la prueba de avidez para IgG, la cual se fundamenta en la medición de la fuerza de interacción entre inmunoglobulinas G específicas y diversos antígenos de *T. gondii.* Estas inmunoglobulinas reconocen epitopos conformacionales o lineales localizados en distintas proteínas del parásito, lo cual permite estimar la maduración de la reacción humoral y, con ello, diferenciar entre infecciones recientes y antiguas, mediante la detección por fluorescencia. Un resultado de poca avidez (≤ 200 UI/ml) indica que se trata de un caso agudo, en el cual la infección se adquirió en los últimos cuatro meses; la avidez intermedia (200 a 300 UI/ml) permite descartar una infección reciente y se sugiere repetir la muestra en 15 dias; y la avidez fuerte (> 300 UI/ml) indica que la infección se encuentra en la fase crónica o que el paciente la adquirió hace más de cuatro meses ^28^.

En la gran mayoría de los casos, el diagnóstico de toxoplasmosis aguda se hace por la presencia de características clínicas típicas de la infección, acompañadas de un perfil serológico compatible y la evidencia de mejoría clinica con el tratamiento antibiótico dirigido.

En la presente serie de casos, se incluyeron cuatro soldados con características clinicas típicas de la infección por *T. gondii* reportadas previamente en la literatura, que se acompañaron de un nexo epidemiológico común (malas condiciones de salubridad y consumo de carne de cerdo cruda) y la confirmación serológica de la toxoplasmosis con contacto reciente con el microorganismo, según la poca avidez reportada; además, se descartaron otras causas potenciales del síndrome febril agudo.

Los hallazgos imagenológicos fueron similares en los cuatro pacientes. La tomografia pulmonar mostró opacidades reticulonodulares, engrosamiento septolobulillar, parches en vidrio esmerilado y derrame pleural, que son los mismos descritos en los reportes de huéspedes inmunocompetentes con compromiso pulmonar ^29^. La mejoria clinica e imagenológica fue completa después de culminar el tratamiento antibiótico con trimetoprim-sulfametoxazol (cuadro 3).

**Cuadro 3:** Características clínicas y de laboratorio de los cuatro casos

Características	Caso 1	Caso 2	Caso 3	Caso 4
Edad	28 años	25 años	28 años	23 años
Sexo	Masculino	Masculino	Masculino	Masculino
Duración de síntomas	10 días	8 días	9 días	8 días
Síntomas	Fiebre, cefalea, mialgias, artralgias, diarrea, disnea y tos no productiva	Fiebre, cefalea, disnea y tos no productiva	Fiebre, cefalea, diarrea, disnea y tos no productiva	Fiebre, alteración del estado de conciencia, disnea y tos no productiva con falla respiratoria hipoxémica
Hepatoesplenomegalia	(-)	(+)	(+)	(+)
Compromiso pulmonar	(+)	(+)	(+)	(+)
Compromiso cardiaco	(-)	(-)	(-)	(+)*
Serología IgG/IgM toxoplasma	(+)	(+)	(+)	(+)
Test de avidez	Baja	Baja	Baja	-
Linfocitosis atípica	14 %	(-)	15 %	8 %
Transaminasas elevadas (AST/ALT)	(+)	(+)	(+)	(+)
LDH	745 U/L	1.113 U/L	1.090 U/L	1.579 U/L
Baciloscopias	Negativas para BAAR	Negativas para BAAR	Negativas para BAAR	-
Serología para dengue	No reactiva	No reactiva	No reactiva	No reactiva
Serología para leptospira	No reactiva	No reactiva	No reactiva	No reactiva
Gota gruesa	Negativa para	Negativa para	Negativa para	Negativa para hemoparásitos
Serología para HIV	hemoparásitos No reactiva	hemoparásitos No reactiva	hemoparásitos No reactiva	No reactiva
Tratamiento	Trimetoprim-	Trimetoprim-	Trimetoprim-	Trimetoprim-sulfametoxazol
Resultado	sulfametoxazol Favorable	sulfametoxazol Favorable	sulfametoxazol Favorable	Favorable

* Disfunción ventricular izquierda (fracción de eyección del 43 %) con troponina cinco veces el limite superior normal y derrame pericárdico escaso

## Conclusiones

Dado que las tropas militares tienen que permanecer largas temporadas en zonas selváticas, están más expuestos a adquirir diversas infecciones tropicales, entre ellas, toxoplasmosis causada por cepas con gran virulencia. Se destaca la heterogeneidad genética de este parásito y su relación con las manifestaciones clínicas de la enfermedad, así como la evidente circulación de cepas selváticas potencialmente letales en la Amazonia colombiana.

Por otra parte, la comunidad médica debe tener en cuenta esta parasitosis como diagnóstico diferencial de un sindrome infeccioso inespecifico con afectación visceral en pacientes con un nexo epidemiológico sospechoso, y apoyarse en el comportamiento clinico y las herramientas diagnósticas complementarias, para indicar de forma oportuna un tratamiento antibiótico dirigido para evitar desenlaces potencialmente fatales.

Este reporte de casos abre la iniciativa a próximos estudios que abarquen el aislamiento y la genotipificación de las cepas circulantes en el Amazonas colombiano, y busca promover estrategias de prevención de esta infección durante la actividad militar, para reconocer y evitar el contacto con fuentes potencialmente contaminadas con este parásito.
